# Chronic dietary exposure to a glyphosate-based herbicide results in total or partial reversibility of plasma oxidative stress, cecal microbiota abundance and short-chain fatty acid composition in broiler hens

**DOI:** 10.3389/fphys.2022.974688

**Published:** 2022-09-12

**Authors:** Mathias Fréville, Anthony Estienne, Christelle Ramé, Gaëlle Lefort, Marine Chahnamian, Christophe Staub, Eric Venturi, Julie Lemarchand, Elise Maximin, Alice Hondelatte, Olivier Zemb, Cécile Canlet, Rodrigo Guabiraba, Pascal Froment, Joëlle Dupont

**Affiliations:** ^1^ Centre National de La Recherche Scientifique, Institut Français du Cheval et de L’Equitation, Institut National de Recherche pour L’Agriculture, L’Alimentation et L’Environnement (INRAE), Université de Tours, Physiologie de La Reproduction et des Comportements, Nouzilly, France; ^2^ INRAE—Unité Expérimentale Pôle D’expérimentation Avicole de Tours, Nouzilly, France; ^3^ INRAE—Unité Expérimentale de Physiologie Animale de L’Orfrasière (UEPAO), Nouzilly, France; ^4^ Université Paris-Saclay, INRAE, AgroParisTech, Micalis Institute, Jouy-en-Josas, France; ^5^ INRAE-—Elevage Alternatif et Santé des Monogastriques (EASM), Surgères, France; ^6^ GenPhySE, Université de Toulouse, INRAE, ENVT, Castanet-Tolosan, France; ^7^ Toxalim (Research Center in Food Toxicology), Université de Toulouse, INRAE, ENVT, INP-Purpan, UPS, Toulouse, France; ^8^ ISP, INRAE, Université de Tours, Nouzilly, France

**Keywords:** birds, glyphosate, metabolism, cecal microbiome, oxidative stress

## Abstract

Glyphosate-based herbicides (GBHs) are massively used in agriculture. However, few studies have investigated the effects of glyphosate-based herbicides on avian species although they are largely exposed via their food. Here, we investigated the potential reversibility of the effects of chronic dietary exposure to glyphosate-based herbicides in broiler hens. For 42 days, we exposed 32-week-old hens to glyphosate-based herbicides via their food (47 mg/kg/day glyphosate equivalent, glyphosate-based herbicides, n = 75) corresponding to half glyphosate’s no-observed-adverse-effect-level in birds. We compared their performance to that of 75 control animals (CT). Both groups (glyphosate-based herbicides and control animals) were then fed for 28 additional days without glyphosate-based herbicides exposure (Ex-glyphosate-based herbicides and Ex-control animals). Glyphosate-based herbicides temporarily increased the plasma glyphosate and AMPA (aminomethylphosphonic acid) concentrations. Glyphosate and aminomethylphosphonic acid mostly accumulated in the liver and to a lesser extent in the leg muscle and abdominal adipose tissue. Glyphosate-based herbicides also temporarily increased the gizzard weight and plasma oxidative stress monitored by TBARS (thiobarbituric acid reactive substances). Glyphosate-based herbicides temporarily decreased the cecal concentrations of propionate, isobutyrate and propionate but acetate and valerate were durably reduced. The cecal microbiome was also durably affected since glyphosate-based herbicides inhibited *Barnesiella* and favored *Alloprevotella*. Body weight, fattening, food intake and feeding behavior as well as plasma lipid and uric acid were unaffected by glyphosate-based herbicides. Taken together, our results show possible disturbances of the cecal microbiota associated with plasma oxidative stress and accumulation of glyphosate in metabolic tissues in response to dietary glyphosate-based herbicides exposure in broiler hens. Luckily, glyphosate-based herbicides at this concentration does not hamper growth and most of the effects on the phenotypes are reversible.

## Introduction

Glyphosate (Gly) is the most widely used herbicide in agriculture worldwide. It is a broad-spectrum herbicide with a generalized effect on all types of crops. Since it is a non-selective product, its commercialization is often coupled with that of genetically modified crops designed to resist the action of the herbicide, enabling farmers to spread increasingly large amounts on their fields without destroying their own crops (Martins-Gomes et al., 2022), while wild plants start to develop natural resistances to it. In animals and plants, Gly is metabolized into CO_2_ and aminomethylphosphonic acid (AMPA) by the enzyme glyphosate oxidoreductase (Mesnage et al., 2015). Gly’s herbicidal effect is due to disruption of the shikimate pathway, which produces aromatic amino acids in plants and in some microorganisms (Schönbrunn et al., 2001). Since humans and animals do not use the shikimate pathway to produce amino acids, Gly is not supposed to have any adverse effect on their health. However, several studies have shown that glyphosate-based herbicide (GBH) formulations can induce tissue damage (Jasper et al., 2012; Larsen et al., 2014), act as endocrine disruptors in various models (Walsh et al., 2000; Romano et al., 2010; Gill et al., 2018), and induce developmental issues in rats brain (Cattani et al., 2021). The use of GBHs is therefore very controversial, since scientific organizations have drawn contrasting conclusions about its dangerousness and recent studies have shown that populations are widely exposed to it (Grau et al., 2022).

Beside their controversial hypothetic effects on human health, GBHs are suspected to have ecotoxicological effects and to be part of the numerous factors leading to the current biodiversity crash. The fate of bird populations is of particular concern. European wild birds are going through a massive decline (Inger et al., 2015) and pesticides (with Gly as the prime representative) are suspected to be major actors of this loss. Gly residues are detectable in soil, water and food (Gill et al., 2018; Fogliatto et al., 2020) which allows them to threaten non-target species. Moreover, it is now established that, like for many pesticides, GBH’s toxicity is enhanced by (if not conditioned to) the presence of other components such as coformulants in herbicide formulations (Bradberry et al., 2004; Kim et al., 2013; Mesnage et al., 2019). Some of them are designed to reduce leaf surface tension and thus to allow penetration of the water-soluble Gly into the plant system first, and then into its cell’s membranes (Mesnage et al., 2019). It is therefore more relevant to study the toxicity of GBHs, rather than that of Gly alone. Few studies of this type have been conducted on poultry, while these animals are frequently exposed to GBHs through their diet. Most studies indeed show that GBHs administered in good farming practice conditions do not have any adverse effect on birds themselves, but rather on their foods and habitats (Gill et al., 2018). However, a recent study shows that GBHs can decrease liver catalase activity and reduce testosterone levels in Japanese quail. The gut microbiome is also disrupted, with a possible suppression of beneficial microorganisms (Ruuskanen et al., 2020). The exposure doses used in this study (12–20 mg Gly/kg body weight/day) being at least five times lower than the NOAEL (no-observed-adverse-effect level; 100 mg Gly/kg body weight/day) reported by the European Food Safety Authority (EFSA), more investigations are needed to characterize the impact of Gly and GBHs on avian models. The host gut microbiome is involved in many processes other than digestion such as xenobiotic detoxication, immune system homeostasis and vitamin synthesis (Samsel and Seneff, 2013). It has been shown that diet composition can induce changes in the gut microbiome bacterial community, and that these changes could have drastic impacts on the host’s biology (metabolism, immunity, behavior etc.) (Sommer and Bäckhed, 2013). Regarding GBHs, a 2013 study demonstrated a reduction of beneficial bacteria in the gastrointestinal tract microbiome after oral administration of GBHs in poultry, while highly pathogenic bacteria were found to be resistant (Shehata et al., 2013). They could therefore induce pathogenic dysbiosis in hens’ gut microbiome.

Thus, the aim of our study was to investigate the effects of a GBH-enriched diet on adult hens’ metabolism and on different biomolecular stress markers, using a dose equivalent to 47 mg Gly/kg body weight/day, half EFSA’s NOAEL, for 6 weeks. We also assessed the reversibility of the potential effects detected, by following the animals for 4 weeks after withdrawal of GBH from their diet. Meanwhile, Gly and AMPA were assayed in plasma and metabolic tissues (leg muscles, liver and abdominal adipose tissue). Detoxication processes were assayed by measuring mRNA expression of the enzymes cytochrome P450 (CYP) and GST (glutathione S-transferase) in animals’ liver, which is the main tissue of biotransformation. We also studied the impact of this diet on gut microbiome composition and diversity. To our knowledge, it is the first study evaluating the potential reversibility of changes in metabolism-related parameters after chronic dietary exposure to a GBH in broiler hens.

## Materials and methods

### Ethical issues

All experimental procedures were performed in accordance with the French National Guidelines for the care and use of animals for research purposes (certificate of authorization to experiment on living animals APAFIS number 21549–2019071809504554v3, approval date: 6 November 2021, Ministry of Agriculture and Fish Products, and a notice of the ethics committee of Val de Loire No. 19).

### Animals

**FIGURE 1:**
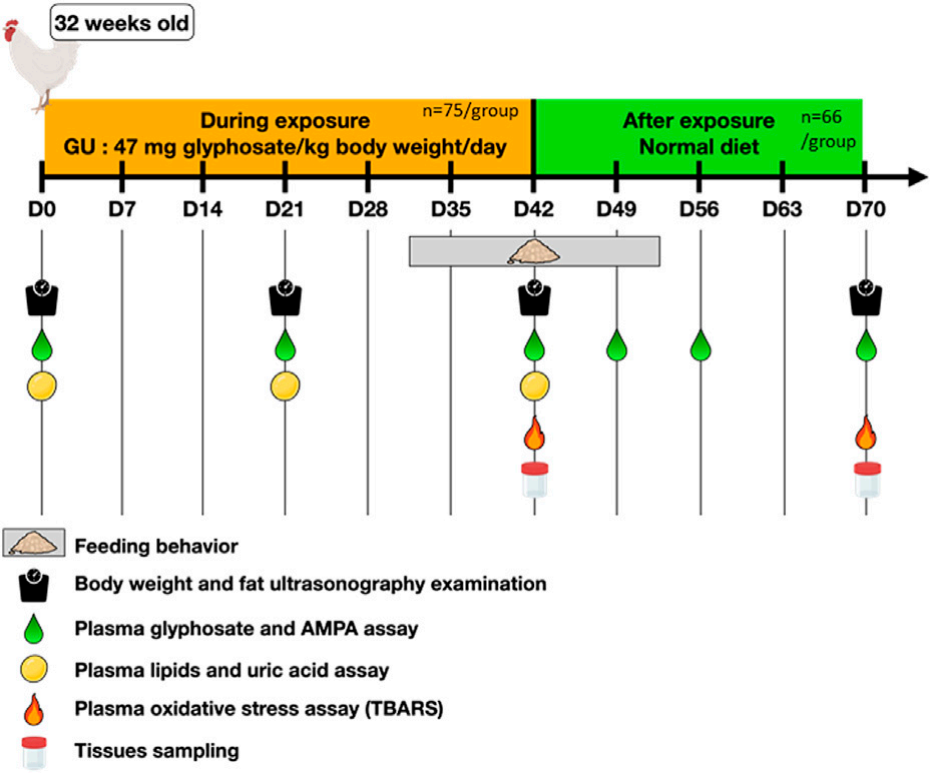
Experimental design applied to hens. Hens (32 weeks old; n = 75) were dietary exposed to GBH (Glyphosate-Based Herbicide, corresponding to a dose of 47 mg glyphosate/kg body weight/day, group named GBH) for 42 days straight, then they received a normal diet (not exposed to GBH, same diet, group named ex GBH) for the 28 following days. Their respective controls (CT, n = 75 and Ex-CT, n = 66) received a normal diet for the whole time. During these 70 days, hens were regularly weighed and ultrascanned to determine fat thickness, their feeding behavior was assayed by counting pecks at the diet and their blood was sampled for glyphosate, AMPA, lipids, uric acid and oxidative stress assays. Nine and twelve animals of both groups were euthanized at D42 and D70, respectively, for tissue sampling.

All animals (150 female chicks of the commercial breed ROSS 308) were obtained at 1 day of age from a local hatchery (Boye Accouvage La Villonniere 79,310 La Boissière en Gatine, France) and reared at “Pôle Expérimental Avicole de Tours” (INRAE, Nouzilly, France) according to traditional breeding conditions. In our experiment, all 150 hens (32 weeks old) were used. They were divided into groups of five birds in 30 pens, each pen with an area of 3 m^2^. The design of the experiment is summarized in Figure 1. The timeline is represented in days. Seventy-five hens (15 pens) were exposed for 42 days to GBH via their food (GBH hens, a GBH dose equivalent to 47 mg Gly/kg body weight/day), and 75 hens (15 pens) were fed with a regular diet without GBH (control animals, CT) (day 0 to day 42 of the protocol). After that, all animals were fed with a regular diet (day 43 to day 70 of the protocol, Ex-GBH and Ex-CT hens). During this protocol, blood samples were collected from hens to quantify Gly and its metabolite AMPA within their blood plasma. At day 42, exposure to GBH was stopped, and nine CT hens and nine GBH hens were slaughtered to recover biological samples. At day 70, 12 Ex-CT hens and 12 Ex-GBH hens were slaughtered to recover biological samples. All animals were killed by electrical stunning and bled out, as recommended by the ethical committee.

### Diet composition

Hens (32 weeks old) received a restricted laying diet according to Hendrix Genetics recommendation. The composition of the diet is shown in Supplemental Table 1. For the GBH animals, this diet was mixed with Gallup super 360 in our laboratory in accordance with the directives of the Directions Départementales de la Protection des Populations (Departmental Directorate for the Protection of Populations). Mixing was carried out by a technician with “Certiphyto” certification for the handling of phytosanitary products, as recommended by French law. Gallup super 360, named GBH within the text, was obtained from Axereal (Monnaie, France); it contained 360 g/L Gly (485.8 g/L isopropylamine salt). Animals were fed with either feed containing GBH (n = 75) or control feed (n = 75) from the first week of the protocol to week 6 (Figure 1). The control feed contained low measurable Gly and AMPA concentrations (0.21 mg/kg feed for Gly and undetectable levels for AMPA as determined by Phytocontrol, Nimes, France). The GBH feed contained 1,250 mg/kg feed of Gly and 0.30 mg/kg feed of AMPA, as determined by Phytocontrol. Hens were food-restricted as recommended by the provider, and their food consumption was 200 g/day. Thus, the concentration in the feed corresponded to a dose of 47 mg Gly equivalent/kg body weight/day. From day 43 to day 70, all hens were fed with control feed (Ex-GBH n = 63 and Ex-CT n = 63).

**TABLE 1:** Oligonucleotide primer sequences used for RT-qPCR.

Tissue	Gene	Primer F	Primer R	References
	*GAPDH*	ACG​GAT​TTG​GTC​GTA​TTG​GG	TGA​TTT​TGG​AGG​ATC​TCG​C	Grandhaye et al. (2021)
	*EEF1α*	AGC​AGA​CTT​TGT​GAC​CTT​GCC	TCA​CAT​GAG​ACA​GAC​GGT​TGC	
	*β-actin*	ACG​GAA​CCA​CAG​TTT​ATC​ATC	GTC​CCA​GTC​TTC​AAC​TAT​ACC	
Liver	*CYP1A1*	AAT​GCT​CGT​TTC​AGT​GCC​TTC	CCT​CCC​CTG​TCC​TTT​TCT​CC	Cong et al. (2019)
	*CYP1A2*	AAC​CCA​GAG​CGT​TTC​CTC​AA	CTC​CCA​CTT​GCC​TAT​GTT​TTC​C	
	*CYP2C*	CAA​AAT​GGA​ACA​GGA​GAA​AGA​GAA​C	CCC​GCA​AGG​AAC​AAG​TCA​A	
	*CYP3A*	CCA​AGC​TAT​GCT​CTT​CAC​CG	TCA​GGC​TCC​ACT​TAC​GGT​CT	
	*CYP2A6*	CTG​CAG​AGA​ATG​GCA​TGA​AG	CCT​GCA​AGA​CTG​CAA​GGA​A	Wang et al. (2018)
	*EPHX1*	GAA​GAT​GTC​AGG​CGG​ATG​TT	CAG​GAG​AGT​CAT​TCA​AAC​CAC​A	
	*GSTA3*	AGA​CCA​GAG​CCA​TCC​TCA​AC	TGC​CAG​TCC​TTC​CAC​ATA​CA	
	*GSTA4*	GCT​ACA​TCG​CAG​GGA​AAT​ACA	TGG​AGA​GAA​AGG​AAA​CAC​CAA	
	*FXR*	AAAGCCGTTCTGTGCGTT	GGATTGGTGGGGTTCCTG	Dai et al. (2020)
	*CYP3A37*	AAA​TCA​GAC​AGC​AAT​GGG​AGC	GGT​AAG​CCA​GGT​AAC​CAA​GTG​T	
	*BSEP*	TGCAAAGCAAAGGAGACT	GCAATGGATAATGGAGGG	
	*CYP1A5*	CTCTGCTCTGTTCDCAAAGCGTCTC	GCTCGCCTGCDCACCDCATCDCACT	
	*CYP1A4*	CDCAGGACGGAGGCTGACDCAAGG	GCDCAGGATGGTGGTGAGGAAGA	
	*Ces1*	TGACCATTCAATATCGCC	ACACTTTCTCCTCCCGCT	
	*SLC O 1B3*	CAG​GAC​TCT​CGC​TGG​GTG​G	TGG​CTT​TCA​GGG​GCT​TTT​T	
	*CYP2H1*	TCA​TCC​ACG​AAA​TCC​AAA​G	GATGGGAGACAGCAAAGG	
	*CYP2H2*	GGCCCGGATGGAGCTATT	TTGCCGCCGAGGTGACTA	
Gizzard	*Hsp27*	ACA​CGA​GGA​GAA​ACA​GGA​TGA​G	ACTGGATGGCTGGCTTGG	Zhao et al. (2016)
	*Hsp40*	GGG​CAT​TCA​ACA​GCA​TAG​A	TTC​ACA​TCC​CCA​AGT​TTA​GG	
	*Hsp60*	AGCCAAAGGGCAGAAATG	TAC​AGC​AAC​AAC​CTG​AAG​ACC	
	*Hsp70*	CGGGCAAGTTTGACCTAA	TTG​GCT​CCC​ACC​CTA​TCT​CT	
	*Hsp90*	TCC​TGT​CCT​GGC​TTT​AGT​TT	AGGTGGCATCTCCTCGGT	
	*NF-κB*	TCA​ACG​CAG​GAC​CTA​AAG​ACA​T	GCA​GAT​AGC​CAA​GTT​CAG​GAT​G	Xing et al. (2015)
	*iNOS*	CCT​GGA​GGT​CCT​GGA​AGA​GT	CCT​GGG​TTT​CAG​AAG​TGG​C	
	*COX-2*	TGT​CCT​TTC​ACT​GCT​TTC​CAT	TTC​CAT​TGC​TGT​GTT​TGA​GGT	
	*PTGES*	GTT​CCT​GTC​ATT​CGC​CTT​CTA​C	CGC​ATC​CTC​TGG​GTT​AGC​A	
	*TNF-*α	GCC​CTT​CCT​GTA​ACC​AGA​TG	ACA​CGA​CAG​CCA​AGT​CAA​CG	
Proventriculus	*PGA5*	TCC​GTC​TAC​CTG​AGC​AAG​GAT	AAG​CAG​GCG​ACG​TAC​TTG​TT	Al-Khalaifah et al. (2020)
	*PGC*	ATC​GGG​ATT​GAG​GAC​TTC​GC	TGA​AGA​CCT​GGT​TGG​GAA​CG	
Caecum	*Chemerin*	CGCGTGGTGAAGGATGTG	CGA​CTG​CTC​CCT​AAA​GAG​GAA​CT	Estienne et al. (2020)
	*CMKLR1*	CGGTCAACGCCATTTGGT	GGG​TAG​GAA​GAT​GTT​GAA​GGA​A	
	*IgA*	GTC​ACC​GTC​ACC​TGG​ACT​ACA	ACC​GAT​GGT​CTC​CTT​CAC​ATC	Grandhaye et al. (2020)
	*IFNα*	CAA​CGA​CAC​CAT​CCT​GGA​CA	GGG​CTG​CTG​AGG​ATT​TTG​AA	Garrido et al. (2018)
	*IFNβ*	TCC​TGC​AAC​CAT​CTT​CGT​CA	CAC​GTC​TTG​TTG​TGG​GCA​AG	
	*IL-1*β	AGG​CTC​AAC​ATT​GCG​CTG​TA	CTT​GTA​GCC​CTT​GAT​GCC​CA	Grandhaye et al. (2020)
	*IL-6*	GCT​TCG​ACG​AGG​AGA​AAT​GC	GCC​AGG​TGC​TTT​GTG​CTG​TA	Garrido et al. (2018)
	*IL-8*	CTG​CGG​TGC​CAG​TGC​ATT​AG	AGC​ACA​CCT​CTC​TTC​CAT​CC	
Spleen	*IFNα*	CAA​CGA​CAC​CAT​CCT​GGA​CA	GGG​CTG​CTG​AGG​ATT​TTG​AA	Garrido et al. (2018)
	*IFNβ*	TCC​TGC​AAC​CAT​CTT​CGT​CA	CAC​GTC​TTG​TTG​TGG​GCA​AG	
	*IL-1*β	AGG​CTC​AAC​ATT​GCG​CTG​TA	CTT​GTA​GCC​CTT​GAT​GCC​CA	Grandhaye et al. (2020)
	*IL-8*	CTG​CGG​TGC​CAG​TGC​ATT​AG	AGC​ACA​CCT​CTC​TTC​CAT​CC	Garrido et al. (2018)
	*IL-22*	TGT​TGT​TGC​TGT​TTC​CCT​CTT​C	CAC​CCC​TGT​CCC​TTT​TGG​A	

### Count of pecks at the diet

Feeding behavior was quantified by counting pecks at the diet over the daily diet distribution. This count was carried out in three hens per pen during the 8 secondes on D31, D32, D35, D36 (during GBH exposure) and D49, D50, D51 and D52 (after exposure).

### Biological samples

Blood samples from 10 hens were collected from the occipital sinus into heparin tubes on different days during the experiment (days 0, 21 and 42 during GBH exposure and days 49, 56 and 70 after exposure). Blood samples were centrifuged (5,000 × *g* for 10 min at 4°C) and stored at −20°C before use for Gly and AMPA assays (Phytocontrol, Nimes, France). Tissue samples were obtained at different points in the experiment [day 42 of the protocol during GBH exposure (n = 9 GBH and n = 9 CT) and day 70 after GBH exposure (n = 12 Ex-GBH and n = 12 Ex-CT)] by dissection after slaughtering.

### Gly and aminomethylphosphonic acid assays in hen plasma

Gly and AMPA concentrations were measured in the blood plasma of hens after a derivatization reaction using FMOC-Cl (9-fluorenylmethyl chloroformate), in collaboration with Dr S El Balkhi (Service de Pharmacologie, Toxicologie et Pharmacovigilance, Limoges, France) as previously described (Serra et al., 2021).

### Lipid and uric acid assays in hen plasma

Plasma concentrations of triglycerides, uric acid, phospholipids and cholesterol were determined by enzymatic assay using specific kits from Biolabo SAS (Maizy, France): triglycerides (reference: LP80519), uric acid (reference: 80,351), phospholipids (reference: 99,105) and cholesterol (reference: 80,106, Biolabo SAS, Maizy, France). The measurements were performed according to the manufacturer’s protocol. For all these assays, the inter- and intra-assay coefficient variations were <15%.

### Body weight gain and measurement of tissue index

Chickens were individually weighed on days 0, 21, 42 and 70. Body weight was recorded and, based on the differences, the body weight gain per day was calculated [(final body weight − initial body weight)/number of days)] for each period D0–D21, D21–D42 and D42–D70 (Figure 1). The weight of organs (liver, spleen, heart, kidney, brain, gizzard) and abdominal adipose tissue (AAT) collected on the 42nd and 70th days of the protocol was determined and the weight of the organs or AAT as a percentage of the body weight was calculated and an organ/tissues index was shown as described by Pandey et al. (2019).

### Plasma thiobarbituric acid reactive substances (TBARS) assay

Lipid peroxidation, as determined through measuring the amount of MDA (malondialdehyde) that reacts with 2-thiobarbituric acid, was used to estimate oxidative stress (Armstrong and Browne, 1994). Blood samples were collected into EDTA-treated tubes, then gently shaken and kept and handled on wet ice. The plasma was separated by centrifuging the blood samples at 1,000 × *g* for 10 min at 4°C, then transferred to 1.5 ml microcentrifuge tubes and stored at −80°C. The TBARS values of the EDTA-treated plasma were measured using the modified method of Grotto et al. (2007). A standard curve for 1,1,3,3-tetramethoxypropane was used, and the concentration was expressed as nmol MDA/mL solution.

### Measurement of liver ATP concentration

Liver total proteins from 9 CT, 9 GBH, 12 Ex-CT and Ex-12 GBH hens were extracted using lysis buffer (1 M Tris (pH 7.4), 0.15 M NaCl, 1.3 mM EDTA, 1 mM EGTA, 43–23 mM VO, 0.1 M NaF, 1% NH_2_PO_4_, 0.5% Triton) and an Ultra-Turrax instrument for grinding, according to the manufacturer’s recommendations (Invitrogen by Life Technologies, Villebon-sur-Yvette, France). Lysates were centrifuged for 20 min at 16,000 × *g* and 4°C, and the supernatants collected. Lysate protein concentrations were then measured using the bicinchoninic acid (BCA) protein assay (Interchim, Montluçon, France). The ATP assay was performed using a Promega CellTiter^®^ Luminescent Cell Viability Assay. Briefly, the assay buffer and the substrate were equilibrated to room temperature, then the buffer was transferred to the substrate and gently mixed with it to obtain a homogeneous solution. After a 30 min equilibration of the cell plate to room temperature, protein lysates (100 µL) were put into a 96-well plate and CellTiter-Glo reagent (100 µL) was added to each well. The plate was orbitally mixed for 2 min and incubated at room temperature for 10 min. The ATP concentration was then measured using a luminometer. Luminescence at the integration time 1,000 (ms) was read using an Ascent Luminoskan Luminometer (Thermo Scientific, Illkirch, France). Lysates’ ATP concentration was normalized with the previously determined total protein concentration.

### Measurement of the gene expression in tissues

Total RNA from 9 CT, 9 GBH, 12 Ex-CT and 12 Ex-GBH hens was extracted from hens’ liver, gizzard, proventriculus, cecum and spleen using TRIzol RNA Isolation Reagents and an Ultra-Turrax instrument for grinding, according to the manufacturer’s recommendations (Invitrogen by Life Technologies, Villebon-sur-Yvette, France). The purity and concentrations of the obtained RNA were checked via their A260/A280 ratios using a Nanodrop machine. cDNA was obtained by reverse transcription of 2 µg of the total RNA in 20 µL of a mix containing each deoxyribonucleotide triphosphate (dATP, dTTP, dGTP, dCTP; 0.5 mM), 2 M RT Buffer, 15 μg/μL oligodT, 0.125 U of ribonuclease inhibitor and 0.05 U of Moloney murine leukemia virus reverse transcriptase (MMLV); the mixture was kept for 1 h at 37°C. Quantitative PCR was performed using a mix of 3 µL of cDNA and 8 µL of SYBR Green Supermix 1X Reagent (Bio-Rad, Marnes-la-Coquette, France) with 250 nM of specific primers (Invitrogen by Life Technologies, Villebon-sur-Yvette, France) given in Table 1. Samples were set up in duplicate in a 384-well plate and a MyiQ Cycle Device (Bio-Rad, Marnes-la-Coquette, France) was used to apply the following procedure: incubation (2 min at 50°C), denaturation (10 min at 95°C) and 40 PCR cycles (30 s at 95°C, 30 s at 60°C, 30 s at 72°C). Relative expression of genes was related to the geometric mean of the expression of three reference genes (*GAPDH* (*glyceraldehyde-3-phosphate dehydrogenase*), *ACTB* (*actin B*) and *EEF1α* (*eukaryotic elongation factor 1 alpha*)). For each target gene, expression was calculated according to primer efficiency (E) and quantification cycle (Cq), where expression = E − Cq. Then, relative expression of the target gene to the three reference genes was analyzed.

### Nuclear magnetic resonance (NMR) metabolomics

^1^H NMR spectra for the metabolic fingerprinting of plasma samples were obtained at 300 K on a Bruker Avance III HD 600 MHz NMR spectrometer (Bruker Biospin, Rheinstetten, Germany) operating at 600.13 MHz for ^1^H resonance frequency using an inverse detection 5 mm ^1^H-^13^C-^15^N-31P cryoprobe attached to a cryoplatform (the pre-amplifier cooling unit). Plasma samples were prepared, and the analyses performed as previously described (Chamorro-García et al., 2021), with slight modifications. For all spectra, a total of 128 transients were collected into 65,536 data points using a spectral width of 20 ppm, a relaxation delay of 5 s and an acquisition time of 2.72 s. All free induction decays were then multiplied by an exponential function with a line broadening factor of 0.3 Hz prior to Fourier transform. All spectra were manually phase- and baseline-corrected, and referenced to the chemical shift of glucose (δ 5.24 ppm).

### Analysis of short-chain fatty acids (SCFAs) in cecal samples

Approximately 35 mg of cecal content was weighed, snap-frozen and stored at −80°C until analysis. The samples were extracted with water and proteins precipitated with phosphotungstic acid. 2-Ethylbutyrate was added to supernatants at a ratio of 1: 4 as an internal standard. The SCFA content was determined from a 0.3 µL volume of supernatant by gas chromatography (Agilent 7890B gas chromatograph, Agilent Technologies, Les Ulis, France) equipped with a split-splitless injector, a flame-ionization detector and a fused silica capillary column (15 m × 0.53 mm × 0.5 µm; Supelco, Saint-Quentin-Fallavier, France). The carrier gas (H_2_) flow rate was 10 ml/min. The oven temperature was initially set at 100°C for 10 min, then increased from 100 to 180°C at a rate of 20 C/min and held for 2 min. The detector temperature was 240°C. Samples were analyzed in duplicate. The peaks obtained were integrated using OpenLAB Chemstation software (Agilent Technologies, Les Ulis, France).

### Microbiome analysis

DNA from bacteria was extracted from cecal content [CT (n = 5), GBH (n = 9), Ex-GBH (n = 10), Ex-CT (n = 5)] using a G’NOME DNA isolation kit (MP Biomedicals, Strasbourg, France) (Furet et al., 2009). The V3–V4 region of the 16S rRNA genes was amplified using MolTaq (Molzym, Plaisir, France), 50 ng DNA and the primers V3F: TACGGRAGGCAGCAG and V4R: ATC​TTA​CCA​GGG​TAT​CTA​ATC​CT (Kozich et al., 2013). Purified amplicons were sequenced using MiSeq sequencing technology (Illumina) on the GeT-PLaGe platform (Toulouse, France). The sequences were submitted to the Short-Read Archive with accession number PRJNA741111. Paired-end reads obtained from MiSeq sequencing were analyzed using the Galaxy-supported pipeline named FROGS (Find, Rapidly, OTUs (Operational Taxonomic Units) with Galaxy Solution) (Escudié et al., 2018). For the preprocessing, reads with a length ≥380 bp were kept. The clustering and chimera removal tools followed the guidelines of FROGS (Escudié et al., 2018). Assignation was performed using SILVA138 16S pintail100. OTUs with abundance lower than 0.005% were removed from the analysis (Bokulich et al., 2013).

### Statistical analysis

GraphPad Prism^®^ software (version 6) was used for all analyses (except for microbiome and NMR metabolomic analysis). All data are reported as means ± standard error of mean (SEM). Outliers were identified by the Rout method and removed. We performed t-tests to compare means at D42 and D70, or two-way ANOVA followed by Tukey’s HSD tests. Regarding gut microbiome analysis, all statistical analyses were performed using R software (version 4.2.0; R Core Team (2022). R: A language and environment for statistical computing. R Foundation for Statistical Computing, Vienna, Austria.). The microbiota composition analysis was performed using the mia package (Ernst et al., 2022). For the variability within bacterial communities ($\alpha$ diversity), Chao, Shannon and Faith indices were computed. The effect of exposure to GBH on $\alpha$ diversity was investigated using Kruskal–Wallis tests followed by Dunn’s post-hoc tests and was considered significant if *p* < 0.05. For the differences between bacterial communities ($\beta$ diversity), the Bray–Curtis matrix and Unifrac distance were computed and then visualized with non-metric multidimensional scaling (NMDS) plots.

For the differential analysis, OTUs with a prevalence below 0.05% were filtered out as well as those with a low number of reads (OTUs with sum counts of less than 0.05% of the sum of all counts). Then, OTU counts were agglomerated at the genus level and relative abundances were computed at each taxonomic level. As recommended by Nearing et al. (2022), three differential analysis methods were used (ALDEx2 (ANOVA-like differential expression 2) (Fernandes et al., 2014), ANCOM-BC (analysis of compositions of microbiomes with bias correction (Lin and Peddada, 2020) and DESeq2 (differential gene expression analysis based on the negative binomial distribution (Love et al., 2014)) and we focused on the common results. All *p*-values were adjusted with the Benjamini–Hochberg correction. A difference is significant if the adjusted *p*-value is below 0.05 and if the size effect is above 1 for ALDEx2 or if the logFC (log fold change) is above 2 for DESeq2. Finally, sparse partial least-squares discriminant analysis (sPLS-DA) was computed on the centered log ratio (CLR)-transformed data using the mixOmics package (Lê Cao et al., 2016; Rohart et al., 2017). The choice of optimal values for the sparsity parameters and the evaluation of classification was performed using a 5-fold cross-validation and 100 repeats.

All analyses were first performed considering all four groups (CT, GBH, Ex-CT and Ex-GBH) separately. Because no significant difference was detected between CT and Ex-CT groups, they were combined and renamed CT + Ex-CT for the microbiome analysis.

## Results

### Chronic dietary glyphosate-based herbicide exposure did not change chickens’ feeding behavior

**FIGURE 2:**
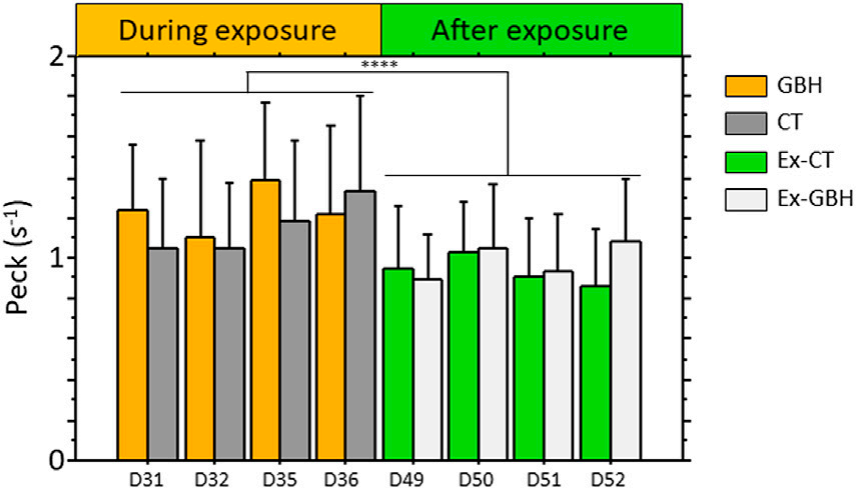
Frequency of pecks for feeding made by control hens (CT, n = 45) and those exposed to GBH (GBH, n = 45) at the manger during (D31, D32, D35 and D36) and after (D49, D50, D51 and D52; Ex-CT, n = 45; Ex-GBH, n = 45) GBH exposure. Results are presented as means ± SEM. ****p ≤ 0.0001 CT: Control, GBH: Glyphosate-Based Herbicide, Ex-CT: Ex-Control, Ex-GBH.

One parameter of the hens’ feeding behavior was estimated by counting the number of times hens pecked at the diet during and after GBH exposure. As shown in Figure 2, no significant difference was detected between GBH and CT hens and between Ex-GBH and Ex-CT hens. All hens made significantly fewer pecks at the diet after exposure than during GBH exposure (*p* < 0.001).

### Chronic dietary glyphosate-based herbicide exposure did not affect chickens’ weight gain but increased gizzard weight

**FIGURE 3:**
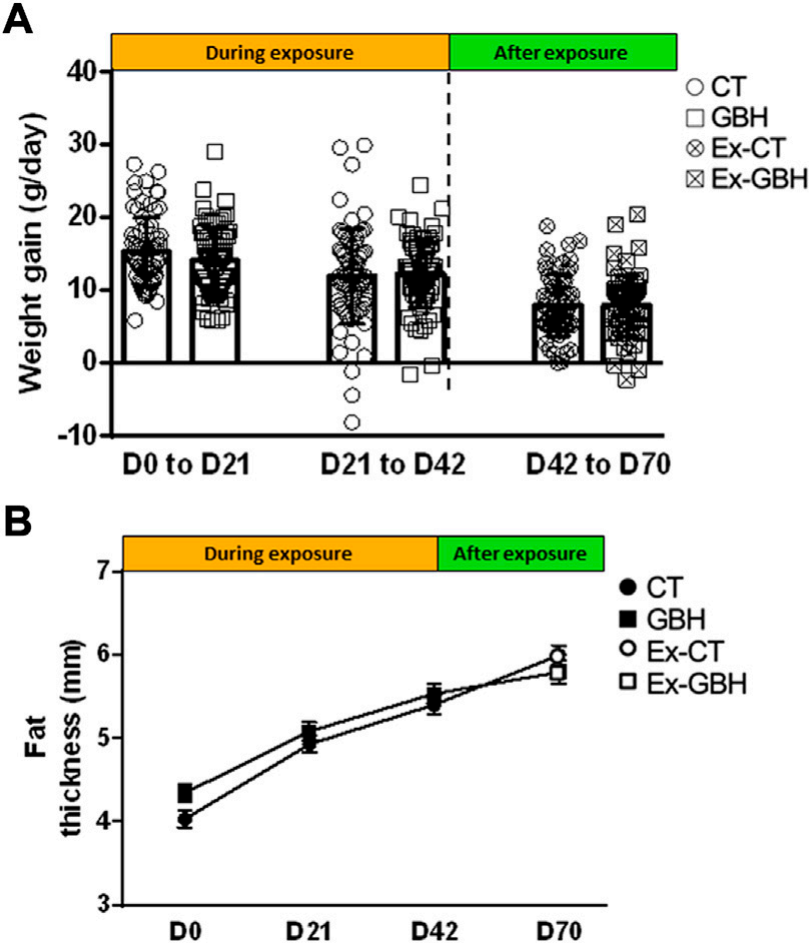
Body weight gained **(A)** and fat thickness **(B)** during dietary GBH exposure (D0 to D42; CT, n = 75; GBH, n = 75) and after (D42 to D70; Ex-CT, n = 66; Ex-GBH, n = 66) dietary GBH exposure. Results are presented as means ± SEM **(A)** and as means **(B)**. CT: Control, GBH: Glyphosate-Based Herbicide, Ex-CT: Ex-Control, Ex-GBH.

Weight gain determined at D0–D21, D21–D42 and D42–D70 is presented in Figure 3A. No significant difference was detected, either during (GBH vs. CT hens) or after GBH exposure (Ex-GBH vs. Ex-CT animals). In addition, no significant effect of GBH on chickens’ fat thickness was detected, either during or after GBH exposure (Figure 3B). Liver, spleen, heart, abdominal adipose tissue (AAT), kidney, brain and gizzard indices are presented in Table 2. During GBH exposure (D42), no significant effect was detected except for gizzards, which were significantly heavier in GBH as compared to CT hens (*p* < 0.05). Furthermore, after exposure (D70), spleens and kidneys were heavier and lighter (*p* < 0.05), respectively, in Ex-GBH hens compared to Ex-CT hens.

**TABLE 2:** Hens organ index [Tissue weight (g)×100)/(Body weight (g)] in GBH exposed and control animals during the period of exposure (42nd day of protocol, D42, CT n = 9, GBH n = 9) and after GBH exposure (70th day of protocol, D70, Ex-CT, n = 12, Ex-GBH, n = 12).

	D42		*p* Value	D70		*p* Value
	CT	GBH		Ex-CT	Ex-GBH	
Liver	1.59	1.68	0.502	1.65	1.64	0.930
Spleen	0.069	0.062	0.318	0.059	0.075*	0.015
Heart	0.360	0.336	0.367	0.359	0.375	0.427
AAT	2.04	2.12	0.734	2.30	2.42	0.148
Kidney	0.154	0.151	0.902	0.377	0.324	0.064
Brain	0.092	0.097	0.097	0.091	0.088	0.667
Gizzard	0.737	0.888***	0.0003	0.812	0.842	0.408

*CT*, control; *GBH, Ex-CT, Ex-Control; Ex-GBH, animals that have been GBH, exposed for 42 days then non-exposed until D70*, Ex-*AAT*, abdominal adipose tissue, Index = (Tissue weight (g)×100)/(Body weight (g)). Results are presented as means. *p ≤ 0.05, **p ≤ 0.01, ***p ≤ 0.001, ****p ≤ 0.0001. *CT, control; GBH, Ex-CT, Ex-Control, Ex-GBH.*

### Gly and aminomethylphosphonic acid accumulated in plasma and tissues after chronic dietary glyphosate-based herbicide exposure in chickens

**FIGURE 4:**
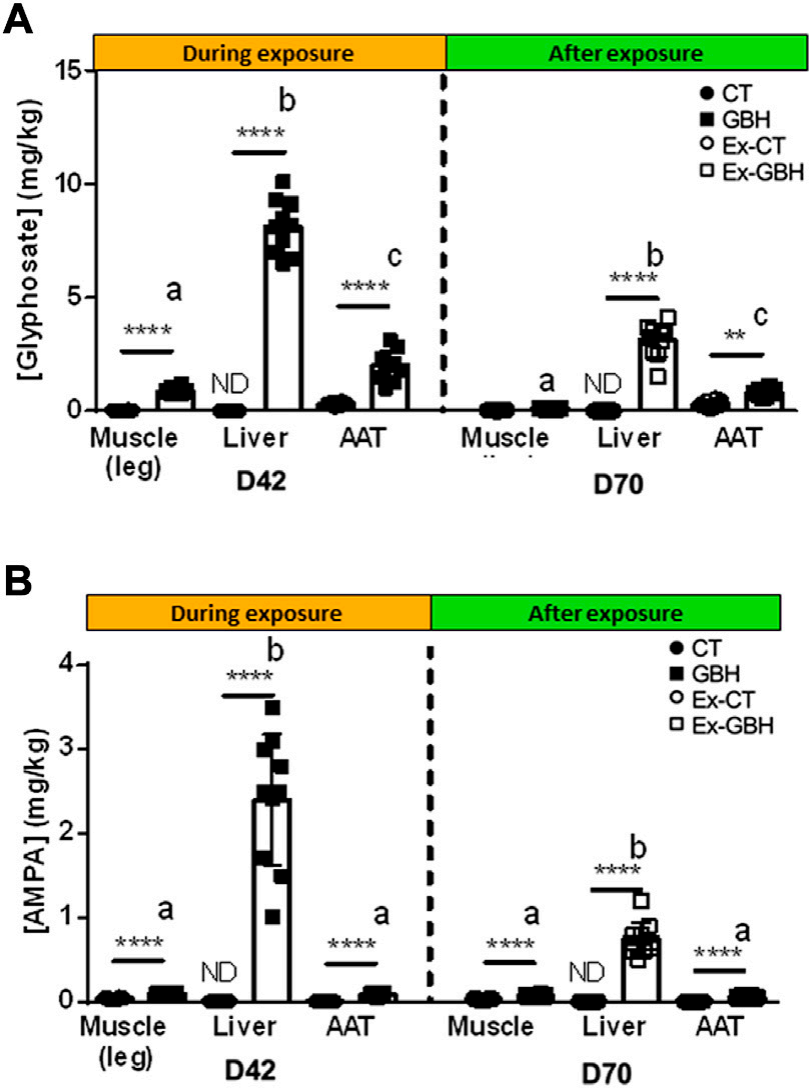
Glyphosate **(A)** and AMPA **(B)** concentrations in hens’ leg muscles, liver and abdominal adipose tissue (AAT) during (D42) and after (D70) GBH exposure. Results are presented as means ± SEM. p-values express mean differences between CT (n = 10) and GBH (n = 10) groups, and Ex-CT (n = 10) and Ex-GBH (n = 10); **p ≤ 0.01, ****p ≤ 0.0001; letters indicate significant differences detected by two-way ANOVA (p < 0.05) followed by Tukey’s HSD test for comparing mean tissue concentrations at D42 and D70 separately. CT: control, GBH: Glyphosate-Based Herbicide, Ex-CT: Ex-Control, Ex-GBH.

Plasma Gly and AMPA concentrations were assayed during GBH exposure and normal diet. The results are presented in Table 3. Plasma Gly and AMPA concentrations reached a peak between D0 and D21 before progressively decreasing to values approximatively twice as low at D42 (1.7- and 1.9-fold decrease, respectively) in GBH animals. Concentrations continued to decrease after GBH exposure. Gly and AMPA accumulations in tissues are presented in Figure 4. Both molecules were found in liver (Gly: 8.10 mg/kg and AMPA: 2.40 mg/kg), AAT (Gly: 2.00 mg/kg and AMPA: 0.13 mg/kg) and leg muscle (Gly: 0.86 mg/kg and AMPA: 0.09 mg/kg) in GBH hens at D42 and were observed in smaller amounts in Ex-GBH at D70 (after GBH exposure): 3.11 and 0.74 mg/kg, 0.80 and 0.04 mg/kg and 0.09 and 0.07 mg/kg in liver, AAT and leg muscle, respectively. In CT and Ex-CT animals, Gly and AMPA were almost undetectable in the three tissues at D42 and D70.

**TABLE 3:** Glyphosate and AMPA concentrations in hen’s plasma during (n = 10) and after (n = 10) the dietary GBH exposure period.

	Time (day)	(Glyphosate] (ng/ml)	(AMPA] (ng/ml)
During exposure	D0	5.37 ± 0.60^a^	0 ± 0^a^
	D21	1,549.02 ± 89.37^d^	19.82 ± 1.75^d^
	D42	910.59 ± 117.29^c^	10.15 ± 0.61^c^
After exposure	D49	297.54 ± 7.96^b^	6.24 ± 0.26^b^
	D56	160.99 ± 9.12^ab^	4.45 ± 1.03^ab^
	D70	67.21 ± 6.87^ab^	1.59 ± 0.52^a^

Results are presented as means ± SEM; letters indicate significant differences detected by Two-way ANOVA, and Tukey HSD’s test for pair-wise comparisons (*p* < 0.05).

### Chronic dietary glyphosate-based herbicide exposure reversibly increased plasma oxidative stress in chickens

**FIGURE 5:**
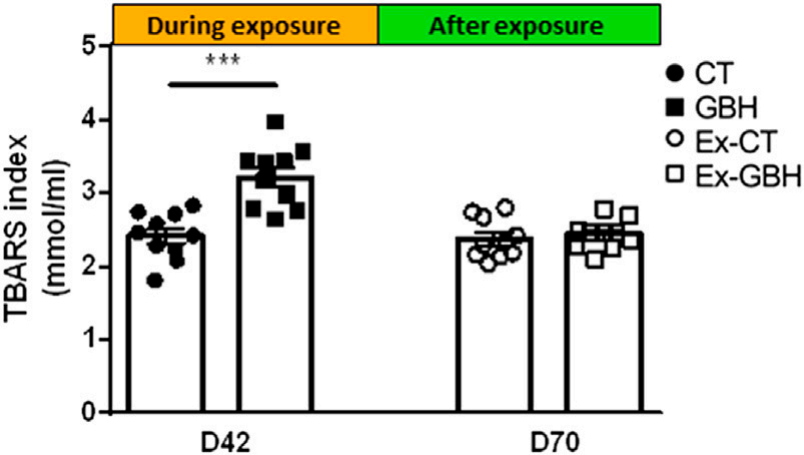
TBARS index in hens’ plasma during (D42; CT, n = 10 and GBH, n = 10) and after (D70; Ex-CT, n = 10 and Ex-GBH, n = 10) GBH exposure. Results are presented as means ± SEM. ***p ≤ 0.001. CT: Control, GBH: Glyphosate-Based Herbicide, Ex-CT: Ex-Control, Ex-GBH.

Plasma oxidative stress was measured using the TBARS index, which quantifies the MDA concentration in plasma. The results are presented in Figure 5. At D42, the TBARS index was significantly (*p* < 0.05) higher in GBH hens than in CT hens. No significant difference was observed between Ex-GBH and Ex-CT hens after GBH exposure at D70.

### Chronic dietary glyphosate-based herbicide exposure did not modulate plasma lipids and uric acid concentrations

Plasma triglyceride, cholesterol, phospholipid and uric acid concentrations were assayed at D0, D21 and D44 (Supplemental Figure S1A–D). No significant effect of GBH exposure on any of these parameters was detected.

### Chronic dietary glyphosate-based herbicide exposure did not trigger biotransformation enzyme transcription in liver but enhanced IgA transcript levels in chickens

The mRNA expression of liver biotransformation enzymes was measured by RT-qPCR. The results are presented in Table 4. No significant effect was detected on any of the transcripts of which we measured the level, either during exposure (CT vs. GBH hens) or after exposure (Ex-CT vs. Ex-GBH). In the cecum, expression of the immunoglobulin A gene (*IgA*) was significantly (*p* < 0.05) enhanced by GBH treatment after exposure in Ex-GBH hens as compared to Ex-CT hens whereas no significant effect was detected during exposure (CT vs. GBH hens). We next determined the expression levels of genes involved in the inflammatory and immune responses. No significant effect was detected for *chemerin*, *CMKLR1*, *IFNα*, *IFNβ*, *IL-1*β, *IL-6* and *IL-8* (Table 4). mRNA expression of stress-related genes in the gizzard was also measured and is shown in Table 4 *iNOS* expression was significantly (*p* < 0.05) higher in the Ex-GBH group than in the Ex-CT group after exposure and that of *HSP70* was significantly (*p* < 0.05) higher in the Ex-CT group than in the Ex-GBH group after exposure. No significant effect on any of these genes was detected during exposure (CT vs. GBH hens). No significant effect on the mRNA expression of some digestive genes (*PGA5* and *PGC*) was detected in the proventriculus (Table 4). The TNF-γ protein concentration in hens’ plasma was assayed and the results are presented in Supplemental Figure 2A. No significant effect of GBH exposure was detected, either at D42 or D70.

**TABLE 4:** Relative mRNA expression of various genes in hen’s organs at the end of GBH exposure (D42, CT, n = 9 and GBH, n = 9) hens) and after dietary GBH exposure (D70, Ex-CT, n = 12 and Ex-GBH, n = 12 hens).

Organ		D42			D70		
		CT	GBH	*p*.value	Ex-CT	Ex-GBH	*p*.value
Liver	*CYP1A2*	10.47	8.74	0.578	16.42	12.31	0.210
	*CYP1A4*	0.110	0.130	0.598	0.170	0.190	0.835
	*CYP2A6*	20.0	18.4	0.696	15.1	16.28	0.741
	*CYP2C*	11.4	9.86	0.498	12.4	11.40	0.793
	*CYP3A37*	6.87	6.78	0.956	5.11	5.16	0.966
	*CYP3A4*	0.300	0.35	0.401	0.35	0.310	0.425
	*CYP3A80*	0.460	0.42	0.681	1.98	0.740*	0.027
	*CYP2H1*	22.8	22.0	0.854	16.7	18.4	0.661
	*CYP2H2*	13.0	13.44	0.905	10.2	12.4	0.432
	*GSTA3*	3.10	2.92	0.700	5.65	3.86	0.200
	*GSTA4*	4.71	3.50	0.054	4.43	4.32	0.861
	*EPHX1*	0.500	0.45	0.541	0.40	0.44	0.763
	*FXR*	0.760	0.78	0.881	1.49	1.24	0.461
	*SL O 1B3*	0.710	0.71	0.972	0.71	0.77	0.759
Gizzard	*COX2*	0.201	0.132	0.188	0.188	0.142	0.194
	*HSP27*	39.9	33.8	0.637	64.1	53.4	0.360
	*HSP40*	94.1	93.1	0.835	91.3	93.3	0.582
	*HSP60*	0.533	0.361	0.113	0.707	0.628	0.310
	*HSP70*	3.18	2.48	0.443	4.31	2.29*	0.015
	*HSP90*	2.24	1.49	0.249	1.87	1.55	0.544
	*iNOS*	0.028	0.022	0.412	0.023	0.042**	0.003
	*NFκB*	0.079	0.059	0.417	0.104	0.099	0.760
	*PTGES*	0.087	0.054	0.168	0.074	0.055	0.245
	*TNFa*	0.036	0.029	0.154	0.023	0.033	0.052
Proventriculus	*PGA5*	32,266	38,158	0.330	26,653	27,223	0.899
	*PGC*	5,916	7,495	0.114	6,874	6,838	0.971
Caecum	*Chemerin*	0.336	0.337	0.986	0.272	0.257	0.731
	*CMKLR1*	0.017	0.017	0.984	0.065	0.048	0.301
	*IgA*	12.3	8.49	0.364	4.46	6.70*	0.041
	*IFNα*	0.071	0.086	0.620	0.434	0.290	0.230
	*IFNβ*	0.002	0.005	0.179	0.024	0.011	0.100
	*IL-1*β	0.013	0.007	0.095	0.020	0.016	0.338
	*IL-6*	0.001	0.001	0.895	0.004	0.002	0.077
	*IL-8*	0.040	0.025	0.185	0.049	0.032	0.242
Spleen	*IFNα*	0.031	0.025	0.607	0.112	0.061	0.108
	*IFNβ*	3.50	2.18	0.301	7.90	6.00	0.414
	*IL-1*β	0.006	0.004	0.182	0.010	0.014	0.279
	*IL-8*	0.076	0.075	0.964	0.065	0.038*	0.036
	*IL-22*	0.035	0.021	0.273	0.093	0.024**	0.003

Results are presented as mean of the pltarget gene mRNA, expression relative to the geometric mean of three housekeeping genes expression (*GAPDH, EEF1α* and *β-actin*); *p ≤ 0.05, **p ≤ 0.01, ***p ≤ 0.001, ****p ≤ 0.0001. *CT, control; GBH, Ex-CT, Ex-Control, Ex-GBH .*

### Chronic dietary glyphosate-based herbicide modified the plasma concentration of glycerol and six amino acids

Metabolites whose concentrations in hens’ plasma were significantly affected by GBH exposure are mentioned in Table 5. Plasma glycerol, isoleucine, lysine, methionine and tyrosine concentrations were significantly increased (*p* < 0.05), while plasma glycine and leucine concentrations were significantly reduced in GBH as compared to CT animals (*p* < 0.05).

**TABLE 5:** Significant plasma metabolites identified in GBH dietary exposed (GBH, n = 10) as compared to control hens (CT, n = 10) at day 42 (D42).

Metabolites	FC	Pvalue
Glycerol	1.05	0.024
Glycine	0.941	0.045
Isoleucine	1.11	0.025
Leucine	0.926	0.024
Lysine	1.53	0.028
Methionine	1.18	0.028
Tyrosine	1.42	0.049

*FC* (Fold Change) = GBH/CT (*CT*, control, *GBH*). Results are presented as means.

### Chronic dietary glyphosate-based herbicide exposure changed SCFA concentrations in chickens’ cecum

Cecal contents were subjected to gas chromatography to measure the cecal SCFA concentration. SCFAs in hens’ cecum were assayed and the results are shown in Table 6. Cecal acetate, propionate, isobutyrate, isovalerate and valerate concentrations were significantly (*p* < 0.05) reduced during exposure in GBH as compared to control animals (CT). All of them were restored in Ex-GBH chickens’ cecum after GBH exposure, as compared to their respective controls (Ex-CT), except for acetate and valerate, which were still significantly (*p* < 0.05) reduced.

**TABLE 6:** Short-chain fatty acid concentration in cecal content (µmol/g) during dietary GBH (GBH, D42, n = 8) exposure in control (CT, n = 8) and after dietary GBH exposure (Ex-CT, D70, n = 12 and Ex-GBH, n = 11 hens).

	D42			D70		
	CT	GBH	*p*.value	Ex-CT	Ex-GBH	*p*.value
Acetate	28.0	18.0**	0.009	25.8	16.6*	0.022
Propionate	8.36	5.43*	0.022	8.02	5.63	0.126
Isobutyrate	0.770	0.510**	0.004	0.730	0.59	0.194
Butyrate	2.08	1.64	0.293	1.85	1.30	0.166
Isovalerate	0.530	0.389*	0.041	0.510	0.450	0.377
Valerate	0.710	0.516*	0.044	0.620	0.390*	0.033
Caproate	0.000	0.000		0.020	0.000	0.068
Total isoAGCC	1.30	0.900**	0.006	1.24	1.04	0.233
Total AGCC	41.5	26.4*	0.015	38.0	25.2*	0.045

Results are presented as means; *p ≤ 0.05, **p ≤ 0.01, ***p ≤ 0.001, ****p ≤ 0.0001. *CT, control; GBH, Ex-CT, Ex-Control, Ex-GBH.*

### Chronic dietary glyphosate-based herbicide exposure increased gut microbiome diversity

**FIGURE 6:**
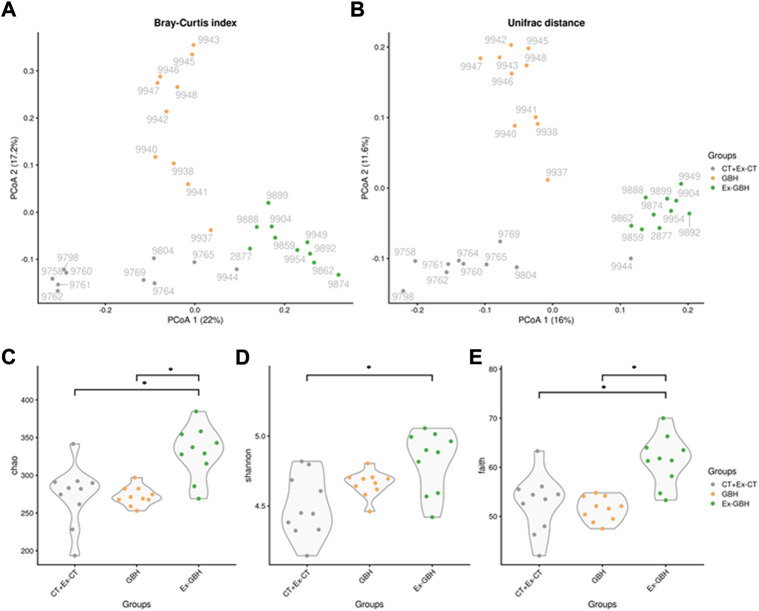
Microbiome β-diversity in cecal content of hens exposed to GBH for 42 days (GBH, n = 9), then non-exposed until D70 (Ex-GBH, n = 12) and controls at D42 and D70 taken together (CT + Ex-CT, n = 10), collected during (D42) and after GBH exposure (D70) measured by principal coordinates analysis (PCoA) on Bray–Curtis index **(A)** and Unifrac distance **(B)**. Results are presented as individual values. Microbiome α-diversity in the very same groups measured by Chao **(C)**, Shannon **(D)** and Faith indices **(E)**. *p ≤ 0.05. CT + Ex-CT: Control and ex-Control taken together, GBH: Glyphosate-Based Herbicide, Ex-GBH.

**FIGURE 7:**
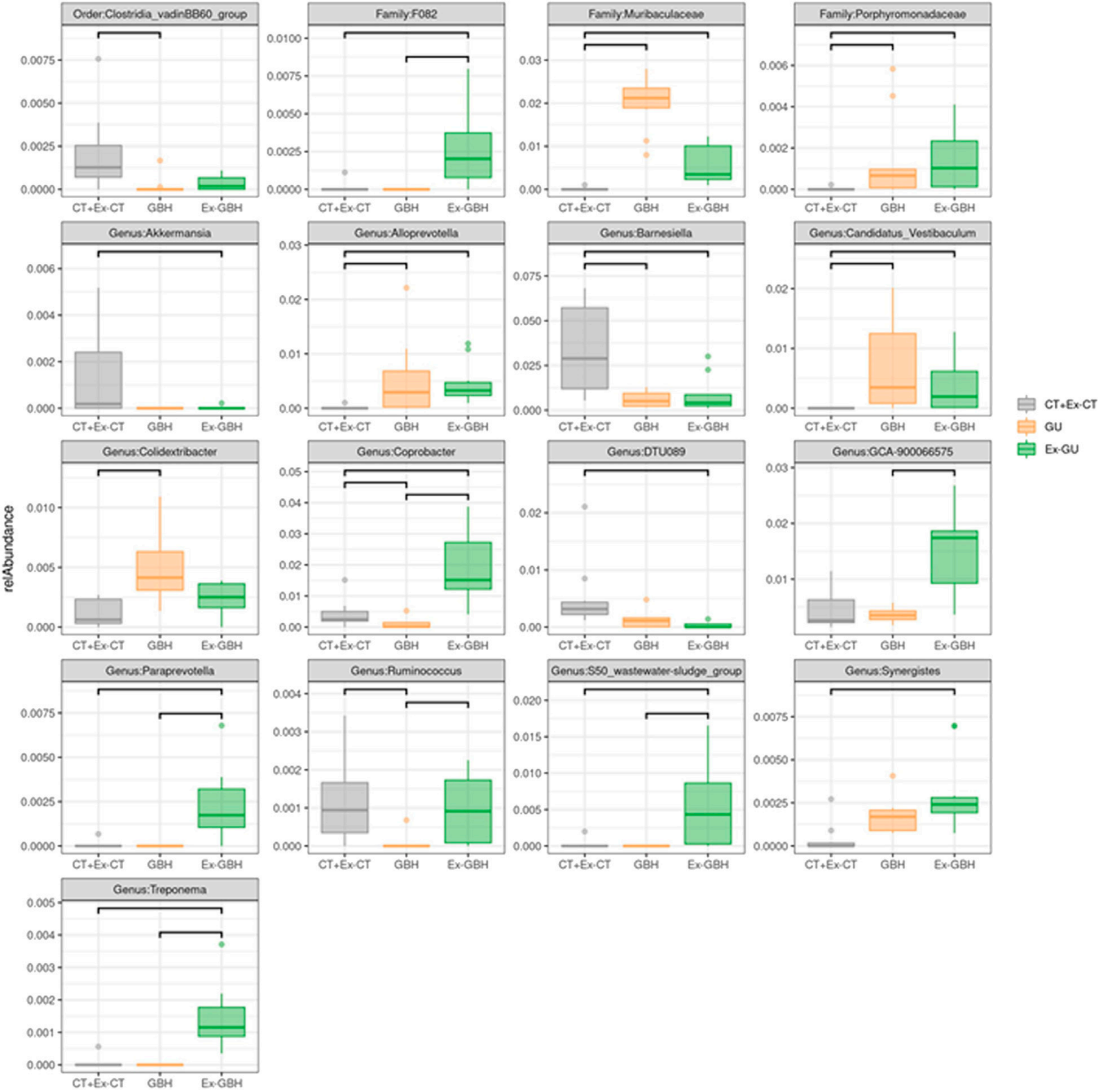
Relative abundance of differential taxa in cecal content of hens exposed to GBH for 42 days (GBH, n = 9), exposed for 42 days then non-exposed until D70 (Ex-GBH, n = 12) and controls at D42 and D70 taken together (CT + Ex-CT, n = 10), commonly identified by ALDEx2, DESeq2 and ANCOM-BC methods. A bar between two groups indicates that the relative abundance is significant between these two groups. Taxa are identified at order, family and genus levels Results are presented as means ± SEM. *p ≤ 0.05. CT + Ex-CT: Control and ex-Control taken together, GBH: Glyphosate-Based Herbicide, Ex-GBH.

GBH durably changed the composition of the microbiota, which was not completely resilient (Figures 6A,B). Surprisingly, GBH increased the diversity of the microbiota in a delayed manner. Indeed, the diversity indices (Chao, Shannon and Faith indices) became different at the final time point even though they were similar at the end of the GBH exposure (Figures 6C–E). The delayed impact of GBH was reflected in eight individual taxa that were different from the control at the end of the experiment but not just after the GBH exposure (Figure 7). For example, the Bacteroidales F082 family was not immediately affected by GBH administration but was significantly enhanced in the Ex-GBH group. Similarly, the *Synergistes* (family Synergistaceae) were favored by GBH at the final time point only. The other groups that showed a delayed impact were: *Akkermansia* (family Akkermansiaceae), *DTU089* (family Clostridiaceae), *Paraprevotella* (family *Paraprevotella*), *S50* (family Rikenellaceae) and *Treponema* (family Treponemataceae). Perhaps unsurprisingly, nine taxa were immediately impacted by GBH: the amounts of Muribaculaceae family, *Alloprevotella* (family Prevotellaceae), Porphyromonadaceae and *Candidatus Vestibaculum* were significantly higher after exposure in GBH and Ex-GBH groups than in the CT + Ex-CT group. The abundance of *Barnesiella* was significantly decreased whereas that of *Colidextribacter* was significantly higher in the GBH group than in the CT + Ex-CT group. The abundance of *Coprobacter* was different in all three groups: it was significantly lower in the GBH group than in the CT + Ex-CT group and was significantly higher in Ex-GBH than in the other groups. *DTU089* abundance was significantly lower in the Ex-GBH group than in the CT + Ex-CT group. *GCA-900066575* abundance was significantly higher in the Ex-GBH group than in GBH hens. *Ruminococcus* abundance was significantly higher in Ex-GBH and CT + Ex-GBH groups than in the GBH group. Abundance of the *S50* wastewater-sludge group was significantly higher in Ex-GBH than in the GBH group. *Synergistes* abundance was significantly higher in Ex-GBH than in the CT + Ex-GBH group. *Clostridia* vadin BB60 abundance was significantly higher in the CT + Ex-CT group than in the GBH group. sPLS-DA analysis was performed to explain group variations. Two components have been identified. Most of the differential taxa identified by sPLS-DA analysis was already identified by the previous analysis. The first component (X-variate 1) explains 16% of the variations and allows to separate GBH and CT + Ex-CT hens from Ex-GBH hens. Second component (X-variate 2) explains 13% of the variations and allows to separate GBH hens from CT + Ex-CT hens. For each component, most contributing taxa were identified based on their abundance. The first component has 5 most contributing taxa. Its most contributing taxon is *Treponema*, more abundant in Ex-GBH group, followed by Prevotellaceae, more abundant in GBH group, *Paraprevotella*, a non-identified *F082* family bacterium, more abundant in Ex-GBH group and *Butyricicoccus* which is more abundant in CT + Ex-CT group. The second component has 5 most contributing taxa. Its most contributing taxon is Muribaculaceae, more abundant in GBH group, followed by *Bacteroidales*, more abundant in GBH group too, itself followed by *Flavobacteriales*, more abundant in CT + Ex-CT group and *Candidatus Vestibculum* and *Synergistes*, more abundant in GBH and CT + Ex-CT group.

## Discussion

**FIGURE 8:**
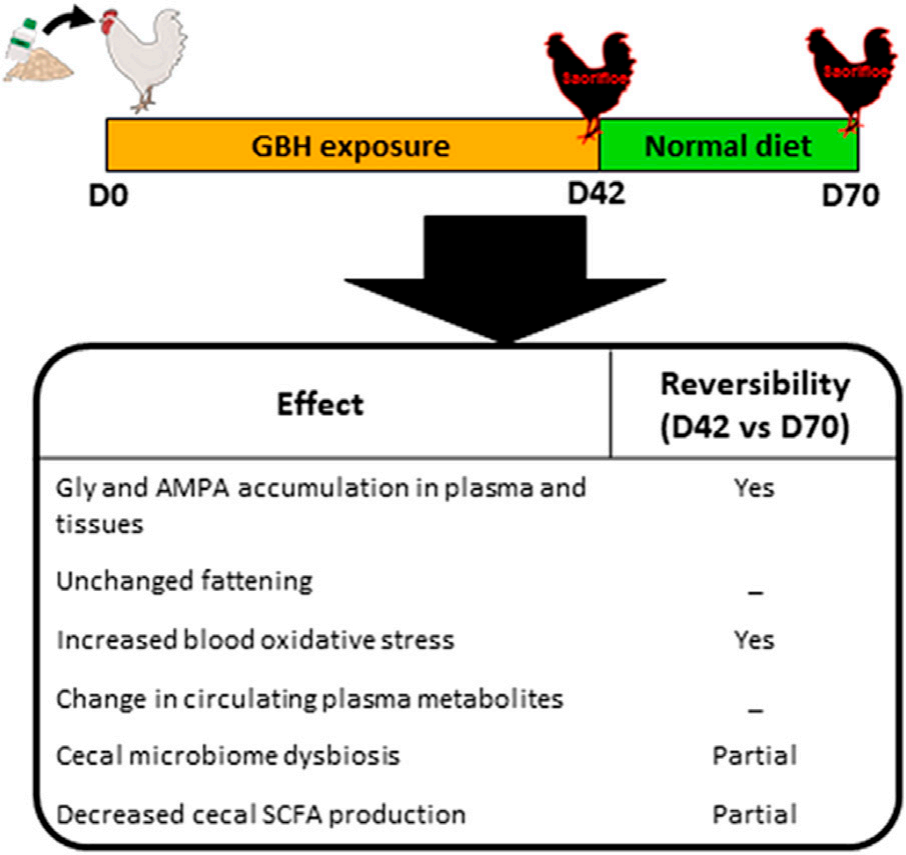
Schematic representation of effects of dietary GBH exposure in hens and their potential reversibility on glyphosate and AMPA accumulation in plasma and tissues, fattening, plasma oxidative stress, plasma metabolites, cecal microbiome dysbiosis and cecal SCFA production.

Our results show for the first time possible disturbances of the cecal microbiota associated with plasma oxidative stress and the accumulation of Gly in metabolic tissues in response to chronic dietary GBH exposure in breeder broiler hens (Figure 8). Furthermore, some of these alterations are potentially reversible and were observed without any variation of growth performance (Figure 8).

Indeed, when we monitored hens’ body weight and fat thickness during and after chronic dietary GBH exposure, we did not observe any differences in either of these two parameters between the hens fed with GBH and those fed without GBH. These results are in accordance with those obtained after a 13-weeks study performed on rats (Panzacchi et al., 2018). Moreover, triglyceride, cholesterol and phospholipid concentrations in plasma were not affected and the uric acid assay did not show any difference with the control either, suggesting that fatty acid and purine metabolism is not affected by the GBH diet. However, metabolomic analysis revealed that the concentrations of some serum metabolites were altered. A recent study shows that in Gly exposed humans, several metabolites levels in serum can be altered (Zhang et al., 2022). These alterations could be linked to metabolism dysfunctions, including fatty acids metabolism and purine biosynthesis. The authors also found that TCA cycle intermediates were altered following to the exposition. In our study, all altered serum metabolites were amino acids (except for glycerol). More specifically, five out of eight of the significantly affected metabolites were ketogenic amino acids, including two glucoformers. Also, all the metabolites we identified were reported several times as potential biomarkers for human colorectal cancer detection (Ni et al., 2014). Among them, leucine, lysine and tyrosine can be oxidized to generate acetyl-CoA for ketone body synthesis; methionine and isoleucine are also convertible into propionyl-CoA, then into succinyl-CoA to enter the TCA cycle (Newsholme et al., 2011; Chiang, 2014; Kumari, 2018). This suggests a potential disruption of mitochondrial activity. However, when we determined the ATP concentration in hens’ livers, we observed no significant effect of the treatment. The effect of such variations in amino acid circulation therefore remains unclear.

Still, it is worth noticing that Gly and AMPA mainly accumulated in the liver and, to a lesser extent, in leg muscles and AAT. However, no significant effect on liver and AAT weight was detected. Gly and AMPA were also detectable in plasma at both D42 (during exposure) and D70 (after exposure), where they were accompanied by an increase in oxidative stress only at D42. Oxidative stress induction has widely been demonstrated in mammals exposed to Gly and AMPA (Kwiatkowska et al., 2014; Owagboriaye et al., 2019; Turkmen et al., 2019). This effect is reversible since plasma TBARS levels drop back to CT values after exposure. The maximum Gly and AMPA concentrations were measured on the 21st day of the diet, and the values measured later on the 42nd day were lower. This suggests that their degradation/excretion rate is lower than their intake rate before D21 but becomes greater during the following days. In order to clarify this observation, we quantified transcript levels of biotransformation enzymes. A recent study has shown that *in ovo* injections of Gly and GBH trigger modulations in the mRNA expression of cytochromes and other biotransformation-related genes in chick embryos (Fathi et al., 2020). Yet, in adult hens, we did not detect any significant effect on the expression of biotransformation-related genes, whether they encode for Phase I (cytochromes) or Phase II enzymes (GSTs). CYP enzymes enable detoxication of xenobiotics by catalyzing redox and hydrolysis reactions, modifying their chemical properties and thus their toxicity. GSTs catalyze the conjugation reaction of GSH to xenobiotics, which enhances their hydrosolubility and thus their elimination in urine and feces. It has been shown that in rats, 98% of administered Gly is excreted as the unchanged parent compound (Panzacchi et al., 2018), which could explain the lack of action of Phase I and Phase II enzymes on it, which would have turned it into modified compounds. Moreover, a 2000 risk assessment reported that orally administered Gly and AMPA are weakly biotransformed in animals (Williams et al., 2000). However, Gly concentrations in the liver do not quickly drop to negligible values after exposure. Yet, as previously mentioned, GBH’s suspected toxicity is not due to Gly alone but also to the surfactants contained in GBH formulations (Bradberry et al., 2004; Kim et al., 2013; Mesnage et al., 2019). Studies have shown that some surfactants are even more toxic than the active ingredient. One example is lipid-based POEAs (polyethoxylated tallow amines) (Martins-Gomes et al., 2022) which are now banned in the EU but still allowed in the United States The presence of these coformulants could explain the high and sustainable accumulation of Gly in the liver, but we would have expected them to trigger the expression of biotransformation enzymes. The exact composition of most GBHs is, however, strictly confidential. We are therefore unable to determine which coformulants could be responsible for this phenomenon.

Considering that hens’ spleens were heavier in the Ex-GBH group than in the Ex-CT group, we assumed a disruption at the immune system level. We therefore investigated immune system gene transcript levels in several immune tissues and observed an increase in IgA mRNA expression in the cecum of Ex-GBH animals compared to that in controls. Since IgA is known to be involved in gut microbiome dynamics (Shi et al., 2017), we suspected potential disruption to hens’ gut microbiome. The main effects of GBH dietary exposure observed in the present study are actually at the gut microbiome level. It is now well established that GBH and Gly are able to induce changes in microbial communities, especially in the gut of exposed animals (Lozano et al., 2018; Mao et al., 2018). A recent study has shown that orally administered Gly and GBH trigger inhibition of the shikimate pathway in the gut microbiome of Sprague Dawley rats. This inhibition is coupled with increased levels of *Akkermansia muciniphila* (Mesnage et al., 2021). Interestingly, our results rather show a decrease in *Akkermansia* abundance, which is not restored after exposure. In humans, the shikimate pathway is mainly achieved by *A. muciniphila* (Mesnage and Antoniou, 2020), which, extrapolated to poultry, could make the crushing of its abundance expectable and consistent since it is the pathway targeted by Gly. Our results also show that *Barnesiella* and *Clostridia* vadin BB60 abundance follows the same scheme, as does that of *Ruminococcus* whose abundance follows a trend toward restoration after Gallup exposure, implicating various potential neuropsychiatric disorders (Barnett et al., 2022). On the other hand, Muribaculaceae, Porphyromonadaceae, *Alloprevotella*, *Candidatus Vestibaculum* and *Colidextribacter* abundances are significantly increased by GBH exposure and follow a trend toward restoration after GBH exposure. Interestingly, *Colidextribacter* species are known to be positively correlated with oxidative stress (Wang et al., 2021). The impact of GBH may be similar between rats and poultry, since GBH exposure also increases *Alloprevotella* in rats’ microbiome (Dechartres et al., 2019). However, to our knowledge, no evident link between variations in the populations of other above-mentioned bacteria and GBH exposure has been established. It has nevertheless previously been shown that a decrease in one bacterial population can induce an increase in other populations (Ruuskanen et al., 2020). We therefore assume that some of the community variations observed here are rather due to inner ecological competition triggered by the reduction in abundance of some taxa than to a direct antibiotic effect of the herbicide. Moreover, Gly is not deleterious to all bacteria: some are able to degrade the molecule (Mesnage and Antoniou, 2020) for phosphorus supply or energy production (Hove-Jensen et al., 2014; Strilbyska et al., 2021). Several recent studies suggest an increase in *α*-diversity in various models when individuals are exposed to glyphosate or GBHs (Tang et al., 2020; Castelli et al., 2021; Mesnage et al., 2021, 2022). Our results are similar to these findings, except that *α*-diversity is not increased in exposed animals, but in formerly exposed animals. It is surprising that adding a perturbation does not have any effect on a system but the removal of this perturbation does produce some significant effects on it. To our knowledge, no previous study has reported this kind of observation following to the administration of dietary GBH. However, since we observe some effects on the gut microbiome at the family and genus levels, we can hypothesize that cecal bacteria quickly accustom to the GBH presence, probably by gaining genetic resistances to it. They could however not accustom to its withdrawal as quickly. Since a genetic resistance, for instance to an antibiotic, is often a disadvantage in absence of it, GBH withdrawal could free up an ecological niche for other bacteria, resulting in an increase in *α*-diversity.

SFCA concentrations in animal feces are also good indicators of potential disruptions in the gut microbiome. Dietary carbohydrates that reach the large intestine without having previously been lysed into smaller molecules in the small intestine are metabolized by bacteria into SCFAs. These molecules are critical in maintaining animals’ health, as they are the primary energy source for colonocytes and are involved in the production of hormones which act on their metabolism (blood glucose regulation, fat and protein digestion, satiation promotion) (Barnett et al., 2022). *Ruminococcus* (Ruminococcaceae) is part of the gut bacteria able to produce SCFAs from dietary carbohydrates (Barnett et al., 2022) that we identified as reversibly diminished by GBH exposure. Our data are also in good agreement with those observed in rats (Dechartres et al., 2019) where Ruminococcaceae abundance was lowered by exposure to GBH. *Akkermansia* has also been reported to be an SCFA (propionate) producer in humans (Morrison and Preston, 2016). Our results show a global diminution of all cecal SCFAs during GBH administration, which are restored after exposure for acetate and valerate only. We can therefore hypothesize a link between the fall of these bacteria and that of cecal SFCA levels. Since SFCAs are also suspected to be involved in neuroendocrine regulation and gut microbiome–brain communication, disturbance in their dynamics suggests potential physiological and mental disorders (Silva et al., 2020). A 2017 study revealed depressive-like behaviors in young adult rats when exposed to a GBH (Cattani et al., 2017). However, we did not detect any effect on hens’ feeding behavior. At this point, it therefore seems important to remember the correlational nature of such studies. Since correlation does not imply causation, more studies are needed to confirm direct guilt of GBH/Gly/surfactants in disruption of the gut microbiome and cecal SFCA production and on the disorders they trigger. Other effects of GBH have been demonstrated in other cells. For instance, it has been shown that low GBH levels could induce oxidative stress and impair Ca^2+^-mediated functions in rat Sertoli cells, resulting in a reduced male fertility (de Liz Oliveira Cavalli et al., 2013). Future works could therefore focus on the potential effects of GBH administration on male and female reproductive functions.

## Conclusion

Here, we show that half the current NOAEL of dietary Gly does not induce a direct impact on hens’ metabolism (at least on the various parameters determined in our 150 animals), though it reversibly triggers oxidative stress in blood plasma and impacts several gut microbes. Investigating the link between GBH and fertility is a direction that future works should probably take, in order to constitute a robust database allowing characterization of the actual impact that the molecule and its formulations have on animal and human health.

## Data Availability

The datasets presented in this study can be found in online repositories. The names of the repository/repositories and accession number(s) can be found below: https://www.ebi.ac.uk/ena, PRJNA741111.

## Glossary

Actin B: Actin Beta ALDEx2: ANOVA-Like Differential Expression 2
AMPA: Amino-Methyl-Phosphonic Acid
ANCOM-BC: Analysis of Compositions of Microbiomes with Bias Correction
BCA: bicinchoninic acid
BSEP: ATP binding cassette subfamily B member 11
Ces1: Carboxylesterase 1
CMKLR1: Chemerin Chemokine-Like Receptor 1
COX-2: Cyclooxygenase 2
Cq: quantification cycle
CT: Control
CYP1A1: Cytochrome P450 Family 1 Subfamily A Member 1
CYP1A2: Cytochrome P450 Family 1 Subfamily A Member 2
CYP1A4: Cytochrome P450 Family 1 Subfamily A Member 4
CYP1A5: Cytochrome P450 Family 1 Subfamily A Member 5
CYP2A6: Cytochrome P450 family 2 subfamily A member 6
CYP2C: Cytochrome P450 Family 2 Subfamily C
CYP2H1: Cytochrome P4502H1
CYP2H2: Cytochrome P4502H2
CYP3A: Cytochrome P450, family 3, subfamily A
CYP3A37: Cytochrome P450, family 3, subfamily A member 37
EPHX1: Epoxide Hydrolase 1
FMOC-Cl: 9-fluorenylmethyl chloroformate
FROGS: Find, Rapidly, OTUs, Operational Taxonomic Units
FXR: Farnesoid X receptor
GAPDH: Glyceraldehyde-3-phosphate dehydrogenase
GBH: Glyphosate-based Herbicides
Gly: Glyphosate
GSH: Glutathione
GSTA3: Glutathione S-Transferase Alpha 3
GSTA4: Glutathione S-Transferase Alpha 4
GSTs: Glutathione S-Transferase
Hsp27: Heat shock protein 27
Hsp40: Heat shock protein 40
Hsp60: Heat shock protein 60
Hsp70: Heat shock protein 70
Hsp90: Heat shock protein 90
IFNα: Interferon alpha
IFNβ: Interferon beta
IgA: Immunoglobulin A
IL-1β: Interleukin-1 beta
IL-22: Interleukin-22
IL-6: Interleukin-6
IL-8: Interleukin-8
iNOS: Inducible nitric oxide synthase
MDA: MalonDiAldehyde
MMLV: Moloney murine leukemia virus reverse transcriptase
NF-κB: Nuclear factor-kappa B
NMDS: non-metric multidimensional scaling
NMR: Nuclear magnetic resonance metabolomics
NOAEL: Non observed adverse effect level
OTUs: Operational Taxonomic Units
PGA5: Pepsinogen A5
PGC: Progastricsin
POEA: Polyethoxylated tallow amines
PTGES: Prostaglandin E Synthase
SCFA: Short-Chain Fatty Acid
TCA cycle*:*: tricarboxylic acid cycle
TNF-α: Tumor Necrosis Factor-alpha
