# Supplementary material for: Chronic dietary exposure to a glyphosate-based herbicide results in total or partial reversibility of plasma oxidative stress, cecal microbiota abundance and short-chain fatty acid composition in broiler hens
Source: Front Physiol. 2022 Sep 12;13:974688. doi: 10.3389/fphys.2022.974688 (PMC9511142; doi:10.3389/fphys.2022.974688)
Supplement: Supplementary file 1 [file Table1.DOCX]

**Supplemental table 1.** Hen’s food composition.

| Component | Percentage |
| --- | --- |
| Corn | 54.19 |
| Wheat | 8 |
| Soybean meal | 13.95 |
| Wheat bran | 0 |
| Soy oil | 2 |
| Sunflower oil | 5.8 |
| Sodium Bicarbonate | 0.1 |
| Calcium carbonate | 6.6 |
| Phosphate | 2.2 |
| Salt | 0.28 |
| Methionine DL | 0.05 |
| Lysine | 0 |
| Mineral premix | 0.5 |
| Soybean | 6.33 |
