# Supplementary figures and images for: Chronic dietary exposure to a glyphosate-based herbicide results in total or partial reversibility of plasma oxidative stress, cecal microbiota abundance and short-chain fatty acid composition in broiler hens
Source: Front Physiol. 2022 Sep 12;13:974688. doi: 10.3389/fphys.2022.974688 (PMC9511142; doi:10.3389/fphys.2022.974688)

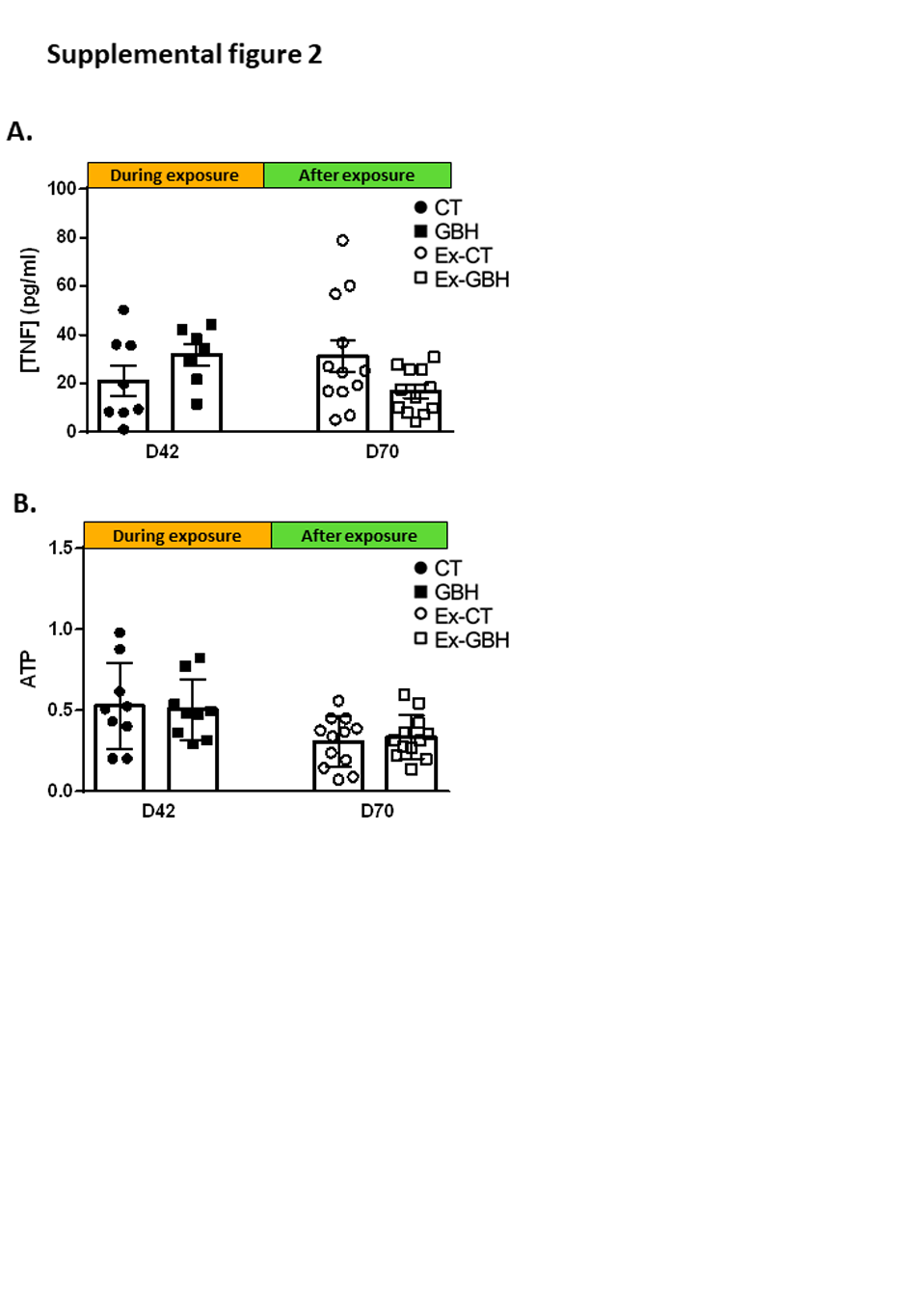

Supplement: Supplementary file 2 [file Image2.TIF]

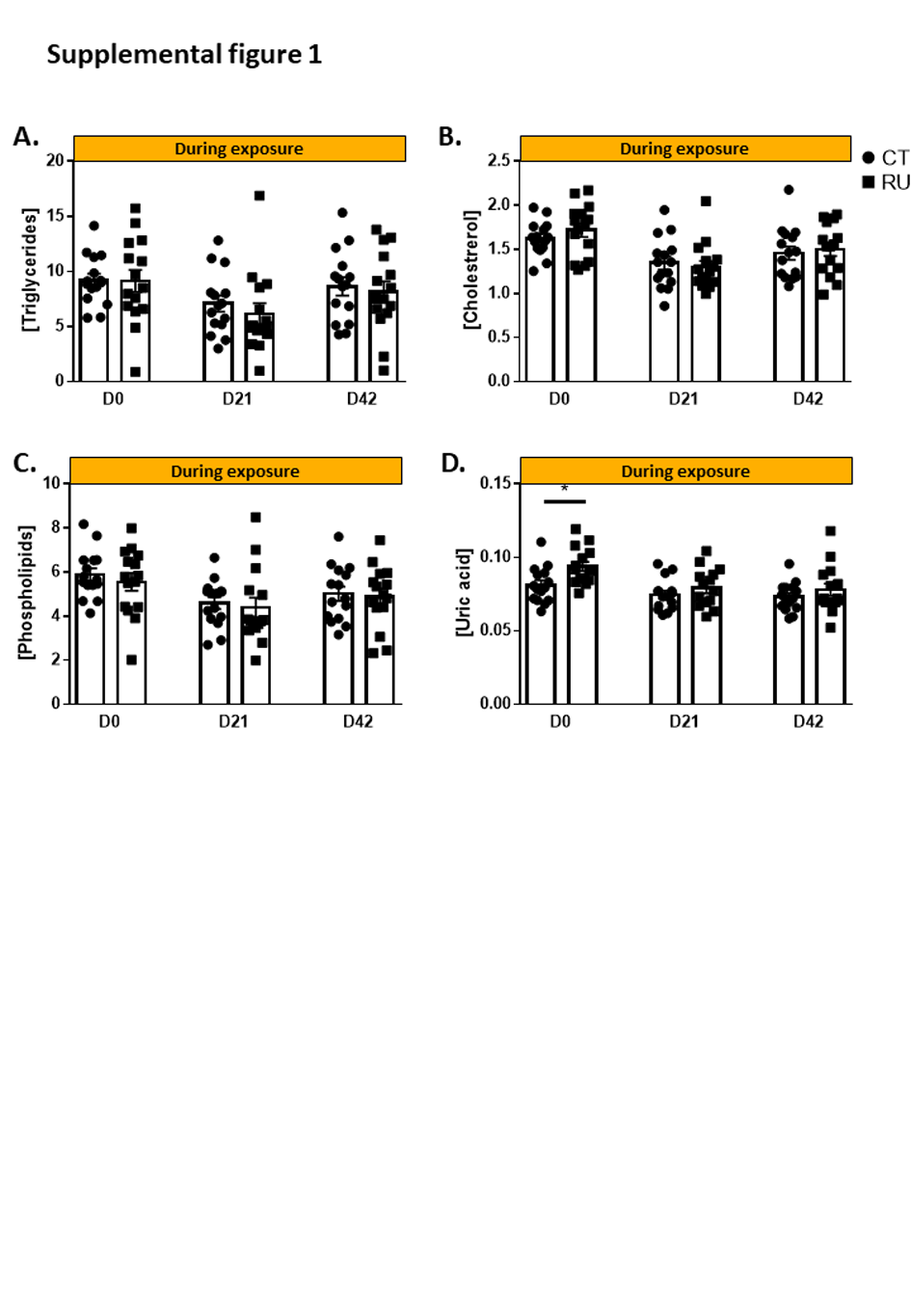

Supplement: Supplementary file 3 [file Image1.TIF]
